# Reevaluating vitamin C in sepsis and septic shock: a potential benefit in severe cases?

**DOI:** 10.3389/fmed.2024.1476242

**Published:** 2024-10-29

**Authors:** Abdulrahman Alissa, Mohammed A. Alrashed, Abdulrahman I. Alshaya, Khalid Al Sulaiman, Shmeylan Alharbi

**Affiliations:** ^1^Pharmaceutical Care Services, King Abdullah Bin Abdulaziz University Hospital, Riyadh, Saudi Arabia; ^2^College of Pharmacy, King Saud Bin Abdulaziz University for Health Sciences, Riyadh, Saudi Arabia; ^3^Pharmaceutical Care Services, King Abdulaziz Medical City, Riyadh, Saudi Arabia; ^4^King Abdullah International Medical Research Center, Riyadh, Saudi Arabia; ^5^Saudi Critical Care Pharmacy Research (SCAPE) Platform, Riyadh, Saudi Arabia; ^6^Saudi Society for Multidisciplinary Research Development and Education (SCAPE Society), Riyadh, Saudi Arabia

**Keywords:** vitamin C, ascorbic acid, sepsis, septic shock, critically ill, pneumonia

## Abstract

Vitamin C (Ascorbic acid) has evolved as an emergent co-intervention for sepsis and septic shock patients. Multiple studies discussed the pathophysiological value of vitamin C to reserve endothelial functionality and improve microcirculatory flow in these patients. Nevertheless, most randomized clinical trials failed to show the clinical impact of adding vitamin C to sepsis and septic shock. Pneumonia is the most common infection to induce sepsis and septic shock, which could be an acute respiratory distress syndrome. Preliminary *in-vitro* data support the role of vitamin C in mitigating the risk of acute respiratory distress syndrome (ARDS) development. This review aims to compare and contrast these trials and explore differences in their patients’ populations, methodologies, and outcomes, emphasizing pneumonia-induced sepsis and septic shock.

## Introduction

Sepsis and septic shock are significant public health burdens with 20–35% predicted mortality rate (1, 2). The pathophysiology of sepsis is a complex interaction of infection and a host that causes a misbalance between pro-inflammatory and anti-inflammatory markers (3–5). The concept of defining sepsis and septic shock has recently moved towards organ dysfunction as the landmark signal for this syndrome based on the Sepsis-3 definition (6). Pneumonia-induced sepsis represents up to 50% of these cases, possibly leading to acute respiratory distress syndrome [ARDS; (7, 8)]. Pneumonia is reported to cause ARDS through a direct insult mechanism in 40–60% of the ARDS patient population (9, 10). During weeks 1–2 of ARDS, an acute inflammatory and exudative phase may lead to endothelial injury and permeability loss. This could progress into endothelial cell death and a fibroproliferative phase, possibly resulting in pulmonary fibrosis (11–13). Substantial evidence points to oxidative stress and dysregulated inflammation as playing a significant role in the onset and development of multiorgan dysfunction and injury in sepsis in both experimental animals and human individuals. Vitamin C has been investigated extensively as a potential treatment for sepsis. *In-vitro* data showed ascorbic acid (or Vitamin C) ability to restore endothelial permeability and improve microcirculatory flow.

Additionally, ascorbic acid is essential for synthesizing catecholamine and enhances vasopressor sensitivity (14–17). Nathens et al. demonstrated ascorbic acid’s ability when used with alpha-tocopherol to reduce the incidence of ARDS and intensive care unit (ICU) lengths of stay in severely ill surgical patients in a randomized prospective trial which was further investigated in other studies (18–23). In the last few years. Multiple randomized controlled trials (RCTs) examined vitamin C role either as a single intervention or as part of a metabolic resuscitation cocktail besides hydrocortisone and thiamin for sepsis and septic patients with inconsistent findings (18, 24–30, 32). Research has shown that administering vitamin C at a dose of 6 g per day is safe and free of side effects, with even higher doses of 100–150 g being safely given to patients with burns or malignancies (31). Intravenous thiamine (vitamin B1) is often combined with vitamin C to prevent potential renal side effects from high doses of vitamin C, while hydrocortisone is used to boost the body’s endogenous production of catecholamines. A recent retrospective before-and-after study reported a significant reduction in mortality among sepsis patients treated with a combination of high-dose vitamin C, hydrocortisone, and thiamine (20). Our primary aim in this narrative review was to focus on the most updated RCTs pertaining to the role of ascorbic acid in sepsis or septic shock, particularly in cases of pneumonia-induced sepsis and septic shock. While the focus on RCTs was a key aspect of our search strategy, we also aimed to provide a comprehensive overview comparing different RCTs based on their patients’ population, methodologies, and outcomes as presented in Table 1.

**Table 1:** Comparison between recent RCTs evaluating the role of vitamin C in sepsis and septic shock patients.

	CITRIS-ALI 2019 (35)	VITAMINS 2020 (32)	HYVCTTSSS 2020 (24)	ORANGES 2020 (27)	ACTS 2020 (29)	Wani 2020 (30)	ATESS 2020 (26)	VICTAS 2021 (25)	LOVIT 2022 (28)
Design	Multicenter, double-blind, placebo RCT	Multicenter, open-label RCT	Single-blind, RCT	Double-blinded, placebo-controlled RCT	Multicenter, blinded RCT	Prospective, open label, single center randomized	Multicenter, double-blind, RCT	Multicenter, double-blind, RCT	Multicenter, RCT
Number of patients	167 patients (84 in vitamin C and 83 in control arm)	216 patients (109 in vitamin C arm and 107 in control arm)	80 patients (40 in each vitamin C arm and 40 in control arm)	137 patients (68 in vitamin C arm vs. 69 in control arm)	200 patients (101 in vitamin C arm vs. 99 in control arm)	100 patients (50 in vitamin C arm and 50 in control arm)	111 patients (53 in vitamin C arm and 58 in control arm)	501 patients (252 in vitamin C arm and 249 in control arm)	872 patients (435 in vitamin C arm and 437 in control arm)
Type of Included patients	Patients with sepsis and ARDS	Septic shock patients (based on Sepsis-3 definition)	Patients with sepsis or septic shock	Patients with sepsis or septic shock	Septic shock patients	Patients with sepsis or septic shock	Septic shock patients (based on Sepsis-3 definition)	Patients with acute respiratory and/or cardiovascular dysfunction caused by suspected infection	Patient with proven or suspected infection and receiving vasopressors
Inclusion criteria	Adults (age ≥ 18 years) diagnosed with sepsis or septic shock according to the Sepsis-3 criteria, ARDS diagnosed according to the Berlin criteria, intubated and mechanically ventilated within 24 h of ARDS diagnosis, randomization within 24 h of meeting ARDS criteria	Adults (age ≥ 18 years) diagnosed with sepsis or septic shock according to the Sepsis-3 criteria, hospitalized in the ICU and requiring vasopressor support with initiation of the trial intervention (vitamin C, thiamine, and hydrocortisone) within 24 h	Adults (age ≥ 18 years) diagnosed with sepsis or septic shock according to the Sepsis-3 criteria, hospitalized in the ICU, procalcitonin (PCT) level ≥ 2 ng/ml and expected to remain in the ICU for at least 48 h.	Adults (age ≥ 18 years) diagnosed with sepsis or septic shock according to the Sepsis-3 criteria, hospitalized in the ICU and requiring vasopressor support with initiation of vitamin C, thiamine, and steroids within 24 h	Adults (age ≥ 18 years) diagnosed with sepsis or septic shock according to the Sepsis-3 criteria, hospitalized in the ICU and requiring vasopressor support with initial serum lactate level > 2 mmol/L after adequate fluid resuscitation.	Adults (age ≥ 18 years) diagnosed with sepsis or septic shock according to the Sepsis-3 criteria, hospitalized in the ICU and requiring vasopressor support with initial serum lactate level > 2 mmol/L after adequate fluid resuscitation.	Adults (age ≥ 18 years) diagnosed with sepsis or septic shock according to the Sepsis-3 criteria, hospitalized in the ICU and requiring vasopressor support, initial serum lactate level > 2 mmol/L after adequate fluid resuscitation with evidence of suspected or confirmed infection	Adults (age ≥ 18 years) diagnosed with sepsis or septic shock according to the Sepsis-3 criteria, hospitalized in the ICU and requiring vasopressor support with initial serum lactate level > 2 mmol/L after adequate fluid resuscitation.	Adults (age ≥ 18 years) diagnosed with sepsis or septic shock according to the Sepsis-3 criteria, hospitalized in the ICU and requiring vasopressor support with initiation of vitamin C within 24 h
Primary outcomes	Change in organ failure by a mSOFA	Duration of time alive and free of vasopressor administration up to day 7	Mortality from any cause within 28 days	Resolution of shock and change in SOFA score.	Change in the SOFA score	In-hospital mortality	Delta SOFA score	Number of consecutive ventilator- and vasopressor-free days in the first 30 days	Composite of death or persistent organ dysfunction on day 28

IV, Intravenous; q, every; h, hours; mSOFA, modified sequential organ failure assessment.

## Clinical studies

Multiple clinical studies investigated the clinical advantages of vitamin C in sepsis and septic shock. Using the keywords “Vitamin C” or “Ascorbic acid” AND “Sepsis” or “Septic Shock” in PubMed, the authors discovered a total of 9 recent RCTs assessing the role of vitamin C in sepsis and septic shock patients. Of the recently published RCTs, two looked at patients with sepsis and compared vitamin C with a placebo. The remaining studies were given either cocktail therapy, which includes hydrocortisone, ascorbic acid, and thiamine (HAT), or standard care as a control group. In this opinion paper, we would like to discuss the rational use of vitamin C and evaluate the current evidence related to the time to intervention, its effect on appropriate dosing of vitamin C, organ dysfunction, and mortality rate.

### Dosing of vitamin C

Most RCTs, including VITAMINS, HYVCTTSSS, ORANGES, ACTS, Wani et al., and VICTAS, adopted the fixed-dose strategy reported by Marik et al. study with 1.5 g of IV ascorbic acid every 6 h for 4 days (20, 24, 25, 27, 29, 30, 32). While other trials, including the most recent one, LOVIT, used weight-based dosing of vitamin C with 50 mg/kg (18, 26, 28). Also, the frequency and duration of ascorbic acid administration varied between the trials that used weight-based dosing. For example, in CITRIS-ALI and LOVIT, vitamin C was given every 6 h for 96 h; in ATESS, the dosing interval was every 12 h for 48 h (18, 26, 28). These vitamin C dosage regimen doses may not be optimal for preventing the pathophysiological processes underlying sepsis. In a meta-analysis, patients with sepsis who received a high dose of vitamin C, defined as more than 50 mg/kg/day, significantly reduced overall mortality (33). However, this positive outcome is contrary to the finding of the most recent and significant RCT, the LOVIT trail, which used a high weight-based dosing of vitamin C and did not report any mortality benefit (28). Surprisingly, the composite outcomes of the LOVIT trial found an increased risk of mortality or persistent organ failure among patients who received vitamin C. Multiple meta-analyses suggest a similar lack of benefit, but several studies remain in progress. The PETAL (Prevention and Early Treatment of Acute Lung Injury) network, supported by funding from the National Heart, Lung, and Blood Institute (NHLBI) made the decision to halt the ASTER (Acetaminophen in Sepsis: Targeted Therapy to Enhance Recovery) study following a series of negative outcomes encountered in multiple studies investigating the role of vitamin C in ARDS. Table 2 summarizes the dosing of vitamin C in sepsis randomized clinical trials.

**Table 2:** Comparison between vitamin C dosing reported by RCTs.

	CITRIS-ALI 2019 (35)	VITAMINS 2020 (32)	HYVCTTSSS 2020 (24)	ORANGES 2020 (27)	ACTS 2020 (29)	Wani 2020 (30)	ATESS 2020 (26)	VICTAS 2021 (25)	LOVIT 2022 (28)
Dose	50 mg/kg every 6 h for 96 h	1.5 g IV every 6 h till shock resolution or up to 10 days	1.5 g IV every 6 h for 4 days or till ICU discharge	1.5 g IV every 6 h for a maximum of 4 days	1.5 g IV every 6 h for 4 days or till ICU discharge	1.5 g IV every 6 h for 4 days or till discharge from hospital	50 mg/kg every 12 h for 48 h (Maximum single dose 3 g, daily dose 6 g)	1.5 g IV every 6 h for up to 96 h, death, or discharge from the ICU, whichever occurred first.	IV vitamin C 50 mg/kg every 6 h for 96 h
Other interventions	None	Hydrocortisone (50 mg every 6 h) and thiamine (200 mg every 12 h)	Hydrocortisone (50 mg every 6 h) and thiamine (200 mg every 12 h)	Hydrocortisone (50 mg every 6 h) and thiamine (200 mg every 12 h)	Hydrocortisone (50 mg every 6 h) and thiamine (100 mg every 6 h)	Hydrocortisone (50 mg every 6 h) and thiamine (200 mg every 6 h)	Thiamine (200 mg every 12 h)	Hydrocortisone (50 mg every 6 h) and thiamine (100 mg every 6 h)	None
Time of intervention	within 6 h of randomization or at earliest available times	12.1 h [5.7–19.0 h]^*^	Not Applicable	9.9 (4.5 h)^**^	14.5 h [8.1–19.1 h]^*^	Within few hours (in all cases in <24 h) of admission	8.4 h [5.7–14.9 h]^*^ from ED arrival and 3.3 h [1.4–7.5 h] ^*^ from randomization	15 h [8–22]^*^	12.9 (8.2)

* Median [IQR].

** Mean (SD).

### Effect of vitamin C on organ dysfunction

The severity of organ dysfunction can be quantified using a SOFA score based on six different systems (34). CITRIS-ALI and LOVIT trials compared vitamin C with a placebo in patients with sepsis and ARDS. Their patients had more advanced organ dysfunction as measured by SOFA score compared with other trials (28, 35). No significant difference was seen in organ dysfunction as a single outcome among patients who received vitamin C compared to the control group from baseline up to 96 h (28, 35). Studies using vitamin C in combination therapy with HAT were inconsistent with their findings. VITAMINS and HYVCTTSS trials reported more significant composite change in SOFA at 72 h (24, 32). In contrast, none of the other RCTs reported a positive impact of the vitamin C intervention on organ dysfunction from baseline up to 96 h (25–30). This suggests that the observed improvement in SOFA scores might be a result independent of the effect of vitamin C. Table 3 summarizes baseline SOFA and change in this score in sepsis RCTs.

**Table 3:** Summary of SOFA and delta SOFA score among RCTs.

	CITRIS-ALI 2019 (35)	VITAMINS 2020 (32)	HYVCTTSSS 2020 (24)	ORANGES 2020 (27)	ACTS 2020 (29)	Wani 2020 (30)	ATESS 2020 (26)	VICTAS 2021 (25)	LOVIT 2022 (28)
Baseline SOFA	9.8 (3.2) in vitamin C vs. 10.3 (3.1) in control group	8.6 (2.7) in vitamin C vs. 8.4 (2.7) in control group	9.6 (4.5) in vitamin C vs. 10.1 (4.0) in control group	8.3 (3) in vitamin C vs. 7.9 (3.5) in control group	9.1 (3.5) in vitamin C vs. 9.2 (3.2) in control group	9.22 (3.54) in vitamin C vs. 9.36 (3.66) in control group	8 [6–10] in vitamin C vs. 8 [6–10] in control group	9 [7–12] in vitamin C vs. 9 [6–11] in control group	10.2 (3.4) in vitamin C vs. 10.1 (3.7) in control group
Δ-SOFA	At 96 h: 6.8 (4.2) in vitamin C vs. 6.8 (3.5) in control group	At 72 h: –2 [−4 to 0] in vitamin C vs. −1 [−3 to 0] in control group [95% CI, −1.9 to −0.1]; *p* = 0.02	At 72 h: 3.5 (3.3) in vitamin C vs. 1.8 (3.0) in control group; *p* = 0 0.02	At 72 h: 2.9 (3.3) in vitamin C vs. 1.93 (3.5) in control group; *p* = 0.1	At 72 h: 4.4 (4.1) in vitamin C vs. 5.1 (4.3) in control group; *p* = 0.12	At 96 h: 5.64 (3.55) in vitamin C vs. 6.62 (3.94) in control group; *p* = 0.20	At 72 h: 3 [−1 to 5] in vitamin C vs. 3 [0–4] in control group; *p* = 0.96	At 96 h: 5 [3–7] in vitamin C vs. 5 [2–7] in control group; *p* = 0.10	At 96 h: 8.7 (6.5) in vitamin C group vs. 8.7 (6.6) in control group

Data reported as mean (SD) or median [IQR]. mSOFA, modified sequential organ failure assessment; IQR, interquartile range; SOFA, sequential organ failure assessment; CI, confidence intervals.

### Effect of vitamin C on mortality

Mortality is a commonly used endpoint in RCTs among critically ill patients (36). In all RCTs that investigated the role of vitamin C, mortality was reported either as primary or secondary outcomes with different duration (37). Out of the RCTs considered, the CITRIS-ALI trial was the only trial that showed a mortality benefit with vitamin C intervention (35). In this trial, 25/84 (29.8%) of vitamin C died by day 28 compared with 38/82 (46.3%) in the control group. However, this finding must be interpreted with caution since the analysis was exploratory and did not account for multiple comparisons. In other RCTs, including HYVCTTSSS, ORANGES, and Wanni et al., a numerically lower mortality percentage was observed among the intervention arm (24, 27, 30). Interestingly, in a subgroup analysis of the ORANGES trial, only patients who were diagnosed within the first 48 h with sepsis showed a significant mortality benefit in the vitamin C group. On the other hand, a numerically more mortality percentage was seen in the vitamin C group in VITAMINS, ACTS, ATESS, VICTAS, and LOVIT trials (25, 26, 28–30, 32). A possible explanation of this could be attributed to the trials’ inclusion criteria, which were mainly septic shock patients or patients with sepsis-induced cardiovascular dysfunction. Additionally, the negative finding of LOVIT trial may be affected by fact that they reported the primary outcome as composite, including death or persistent of orang dysfunction (28). The results of the LOVIT trial showed an implicit lack of benefit and an increase in mortality risks associated with the administration of large dosages of vitamin C. The effect of vitamin C on mortality among sepsis RCTs are summarized in Table 4.

**Table 4:** Summary of vitamin C effect on mortality as reported by RCTs.

	CITRIS-ALI 2019 (35)	VITAMINS 2020 (32)	HYVCTTSSS 2020 (24)	ORANGES 2020 (27)	ACTS 2020 (29)	Wani 2020 (30)	ATESS 2020 (26)	VICTAS 2021 (25)	LOVIT 2022 (28)
28 or 30 days mortality	29.8% in vitamin C vs. 46.3% in control *p* = 0.03	22.6% in vitamin C vs. 20.4% in control	27.5% in vitamin C vs. 35% in control^*^	NA	34.7% in vitamin C vs. 29.3% in control	40% in vitamin C vs. 42% in control	20% in vitamin C vs. 15.5% in control	NA	35.4% in vitamin C vs. 31.6% in control
ICU mortality	NA	19.6% in vitamin C vs. 18.3% in control	NA	9% in vitamin C vs. 14% in control	NA	NA	15.2% in vitamin C vs. 13.5% in control	20.6% in vitamin C vs. 19.7% in control	NA
Hospital mortality	NA	23.4% in vitamin C vs. 20.4% in control	NA	16% in vitamin C vs. 19.4% in control	NA	24% in vitamin C vs. 28% in control	24.5% in vitamin C vs. 19% in control	NA	NA
90 days mortality	NA	28.6% in vitamin C vs. 24.5% in control	NA	NA	NA	NA	32.1% in vitamin C vs. 27.6% in control	40.5% in vitamin C vs. 37.8% in control^**^	NA

* In subgroup analysis, patients who diagnosed with sepsis within 48 h showed a lower mortality (*p* = 0.02).

** 180 days mortality.

### Effect of vitamin C on mortality in sepsis and septic shock

The severity of sepsis and advanced organ dysfunction could affect mortality (6, 36). Among different sepsis RCTs, the percentage of septic shock patients varied between studies and ranging from 55 to 100% (18, 24–30, 32). Some trials, including HYVCTTSSS and CITRIS ALI, included patients with sepsis and septic shock, while other trials like ORANGES and LOVIT have targeted septic shock patients as inclusion criteria (18, 24, 27, 28). Regarding the reduction of vasopressors duration, the same conflicting findings were observed among the published RCT. In ORANGES and Wani et al. trial, the use of vitamin C has led to a significant reduction in vasopressor duration (27, 30). Also, the ACTS trial showed more shock-free days among patients assigned to vitamin C group (29). These data could point towards a potential benefit for vitamin C in patients with early signs of sepsis. Table 5 summarized the factors that may affect mortality.

**Table 5:** Major factors that may affect overall mortality in sepsis and septic shock.

	CITRIS-ALI 2019 (35)	VITAMINS 2020 (32)	HYVCTTSSS 2020 (24)	ORANGES 2020 (27)	ACTS 2020 (29)	Wani 2020 (30)	ATESS 2020 (26)	VICTAS 2021 (25)	LOVIT 2022 (28)
APACHE II or III Scores	NA	Vitamin C: 77.4 (29.7)^*^ Control: 83.3(28.8)	Vitamin C: 22.1 (8.4) Control: 23.8 (7.6)	Vitamin C: 24 (7.6) Control: 24.9 (8.7)	NA	Vitamin C: 18.5 [15–24.75] Control: 20 [14–24]	Vitamin C:22 [14–32] Control: 22 [17–32]	Vitamin C: 27 [22–33] Control: 27 [19–33]	Vitamin C: 24.2 (7.4) Control:24.1 (7.9)
Vasopressor use	Vitamin C 64.3% Control: 71.1%	Vitamin C: 100% Control: 100%	Vitamin C: 55% Control: 60%	Vitamin C: 82% Control: 68%	Vitamin C: 100% Control: 100%	Vitamin C: 92% Control: 76%	Vitamin C: 100% Control: 100%	Vitamin C: 36.9% Control: 39.1%	Vitamin C: 99.8% Control: 100%
Steroids administration	Vitamin C: 67% Control: 65%	Vitamin C: 42.1% Control: 37.5%	N/A	Steroid was part of intervention group who received vitamin C Control: 41%	Steroid was part of intervention group who received vitamin C Control: 14.1%	N/A	Vitamin C: 58.5% Control: 50%	Vitamin C:33% Control: 32%	Vitamin C: 46.4% Control:45.4%

Data reported as mean (SD) or median [IQR].

Several studies suggested a potential benefit for vitamin C to prevent or treat pneumonia (19, 37, 38). These patients may express superimposed inflammatory responses, which ascorbic acid may be able to mitigate. In HYVCTTSSS, CITRIS-ALI, and Wani et al., pulmonary infections were the most predominant types of infections ranging at least 72–82% of patients included in these trials, followed by urinary infection gastrointestinal, respectively (18, 30, 32). Both of HYVCTTSSS, and CITRIS-ALI reported a lower mortality with the administration of vitamin C (18, 24). In VITAMINS, ACTS, and LOVIT trials, the primary infection site, in patients who received vitamin C, was almost equally shared between pulmonary and gastrointestinal infections, which could have diluted the potential effect of vitamin C (29, 32). Moreover, unlike the previous trials, the suspected source of sepsis was an intra-abdominal infection in 50.9% of cases in the ATESS trial followed by respiratory and urinary tract infections, which did not have beneficial clinical outcomes with the administration of vitamin C (26). It is hard to correlate this mortality benefit to the subset of patients who developed pneumonia-induced sepsis or septic shock; however, this observation may necessitate further investigation. In the subgroup analysis of LOVIT trial, a trend towards benefits of vitamin C was observed among patients with severe acute respiratory syndrome coronavirus 2 (COVID-19) although it was not clinically significant (28). In most of the aforementioned sepsis randomized clinical trials, the administration of vitamin C was not associated with mortality benefit (24–30, 32). Sepsis and septic shock mortality could be attributed to multiple interventions such as the timing of antibiotics from disease onset, appropriate selection of antibiotics, appropriate volume resuscitation and de-resuscitation, vasopressor administration, and corticosteroids administration (39–43). Mostly vitamin C administration was combined with hydrocortisone 50 mg every 6 h in the intervention arm as part of hydrocortisone, ascorbic acid, thiamine, or (HAT) protocol (20, 25, 32). For instance, 67% received corticosteroids in the CITRIS-ALI trial, and 58.5% received corticosteroids in the ATESS trial (26, 32). Among all RCTs, the resolution of shock was observed in two trials only (27, 30). In the ORANGES trial, the time of shock reversal was 27 ± 22 h in vitamin C arm compared with 53 ± 38 h in the control arm (27). Additionally, in Wani et al. trial, the duration of vasopressors was 75.2 ± 30.2 h in the vitamin C group compared with 96.1 ± 40.5 in the control group (30). Likewise, the use of vitamin C was associated with significant shock-free days compared with placebo in ACTS trial; however, this finding was not replicated in VITAMINS nor HYVCTTSSS trials (24, 27, 30). Moreover, recently a systematic review and meta-analysis included 18 RCTs that showed the use of IV vitamin C in septic shock patients was associated with better delta SOFA scores but did not show a decrease in short-term mortality (44).

### Precision medicine

Sepsis is a complex syndrome with a broad spectrum of conditions, severities, and clinical presentations. Standardized treatments often fall short of addressing the diverse needs of all patients, underscoring the necessity for targeted, individualized therapies. The heterogeneity of sepsis—shaped by factors such as patient age, causative microorganisms, infection type, and pre-existing conditions—highlights the need for precision medicine approaches that tailor treatment to the unique clinical and biological characteristics of each patient.

Recent research has increasingly explored the potential benefits of high-dose vitamin C, often administered in combination with hydrocortisone and thiamine, commonly referred to as a “sepsis cocktail.” This combination has been proposed to reduce mortality, decrease reliance on vasopressors, and mitigate organ damage in septic patients. However, RCTs have produced inconsistent results on the efficacy of vitamin C in improving clinical outcomes. For example, the CITRIS-ALI trial suggested a potential mortality benefit, while other studies reported no benefit and even showed potential harm, such as increased risk of mortality or persistent organ failure with high-dose vitamin C. Although some trials have shown improvements in SOFA scores with vitamin C, these benefits were not uniformly seen, suggesting that other factors may also contribute to organ function recovery. It is important to note that the SOFA score may not capture all dimensions of treatment outcomes and might overlook certain effects of interventions. The variability in dosing strategies and outcomes across different RCTs further emphasizes the need for personalized treatment approaches. While some studies have shown benefits with fixed or higher doses of vitamin C, others, like the LOVIT trial, found no mortality benefit and even suggested potential harm with high-dose vitamin C.

These mixed results from RCTs highlight the limitations of a one-size-fits-all approach in sepsis management. Precision medicine, which emphasizes individualized treatment strategies, offers a promising pathway to improving outcomes for critically ill patients. By focusing on early identification and targeted intervention, precision medicine aims to enhance the effectiveness of therapies like vitamin C, especially for patient subgroups most likely to benefit. Advancing our understanding of patient-specific factors and refining treatment protocols could play a pivotal role in improving care for patients with sepsis and septic shock.

## Conclusion

The co-administration of vitamin C with sepsis and septic shock patients did not consistently demonstrate a mortality benefit. However, our review suggests a potential mortality advantage of vitamin C co-administration, especially in patients with advanced disease severity when initiated early in sepsis or septic shock therapy. A subgroup analysis highlights the possibility of a beneficial effect in critically ill patients developing sepsis or septic shock secondary to pneumonia, indicating a potential area for further research. It’s noteworthy that the observed improvement in SOFA scores raises questions about whether this improvement is independent of the effect of vitamin C. Although several well-designed randomized clinical trials have investigated the role of vitamin C in sepsis, further data and research are warranted to confirm and elucidate these findings.
